# The complete mitochondrial genome of *Percocypris retrodorsalis* (Teleostei, Cypriniformes) in Nujiang River: characterization and phylogenetic position

**DOI:** 10.1080/23802359.2020.1821815

**Published:** 2020-09-17

**Authors:** Xiao-Qin Xiong, Xing-Jian Yue

**Affiliations:** a Key Laboratory of Sichuan Province for Fishes Conservation and Utilization in the Upper Reaches of the Yangtze River, College of Life Sciences, Neijiang Normal University, Neijiang, P. R. China; b College of Life Science, Neijiang Normal University, Neijiang, P. R. China

**Keywords:** Cyprinidae, *Percocypris retrodorsalis*, mitogenome, phylogenetic analyses

## Abstract

*Percocypris retrodorsalis* is an endemic species found in Nujiang River and Lantsang. In this study, the complete mitochondrial genome of *P. retrodorsalis* was determined. The circular mitochondrial genome was 16,576 bp long, containing 13 protein-coding genes, two ribosomal *RNA* genes (rRNA), 22 transfer RNA (*tRNA*) genes, an origin of light-strand replication (OL), and one displacement loop locus (D-loop). Most genes were encoded on the heavy strand except for ND6 and eight *tRNA* genes. There were 11 regions of gene overlaps totaling 29 bp and seven intergenic spacer regions totaling 37 bp. The phylogenetic analyses were performed on the concatenated dataset of 28 protein-coding genes (PCGs), and the fishes of genus *Percocypris* may have a close relationship with Schizothoracins (Schizothoracinae) compared to other Cyprinidae fishes.

The genus *Percocypris* (Barbinae, Cyprinidae, and Teleostei) is a group of fierce predatory freshwater fishes inhabiting large rivers or lakes in southwestern China and northern Vietnam (Yue 2000; Wang et al. 2013). The species of *Percocypris* in Nujiang River was controversial and was considered to be subspecies or independent species (Cui and Chu 1990; Kottelat 2001). The molecular phylogenetic relationships based on three mitochondrial genes (*16S*, *COI*, and *Cyt b*) and one nuclear marker (Rag2) advised that the species of *Percocypris* in Nujiang River should be treated as a separate species (Wang et al. 2013). Here, this species was identified as a full species followed the classification system of Chen (2013) and Zhang et al. (2016).

*Percocypris retrodorsalis* is an endemic economic fish species found in Nujiang River (the upper Salween River basin) and Lantsang River (the upper Mekong basin). It was occasional species in Nujiang River (Liu et al. 2016). It has become endangered in recent years due to overfishing and has been listed as a near-threatened species due to its great individual, high economic value, narrow distribution, and small resources (Liu et al. 2016). There were complete mitochondrial genomes of *Percocypris pingi* in the Yangtze basin in China (GenBank accession number JX316026) (Li et al. 2013). However, the available genetic data are still scarce for this species. In this study, we sequenced the complete mitochondrial genome sequence of *P. retrodorsalis* using Sanger method. The mitogenome sequence data of *P. retrodorsalis* presented in this work would provide the fundamental genetic data for inferring phylogenetic relationships of *Percocypris* and conservation genetic studies for this species.

The samples of *P. retrodorsalis* were collected from Nujiang River in Shangjiang Town, Lushui Country, Yunnan Province, China (98°53′16′′ E, 28°38′15′′ N) and were stored at Yangtze River Fisheries Research Institute (No.20071203003). The total genomic DNA was extracted from the fin tissue using the salt-extracted method (Aljanabi and Martinez 1997) with some modifications. Twenty-one pairs of PCR primers were designed to amplify and sequence the complete mitochondrial genome. Eight new middle primers were designed for sequencing, if entire target segments are not obtained by using the aforementioned primers. The overlapping segments were analyzed using the software Lasergene V11 (DNASTAR) and Mega X (Kumar et al. 2018). The mitogenome was annotated with the MITOS (http://mitos2.bioinf.uni-leipzig.de/index.py; Bernt et al. 2013), combined with manual corrections.

The total length of *P. retrodorsalis* circular mitogenome is 16,576 bp. This mitogenome was submitted to GenBank database with accession No. MT527960. The overall base composition of the genome is as follows: A (31.2%), T (25.2%), C (27.1%), and G (16.4%) with a slight AT bias of 56.4%. It was an average AT bias rich feature of teleost mitochondrial genomes.

Most mitochondrial genes were encoded on the heavy strand except for ND6 and eight tRNA genes (*tRNA^Gln^*, *tRNA^Ala^*, *tRNA^Asn^*, *tRNA^Cys^*, *tRNA^Tyr^*, *tRNA^Ser(UCN)^*, *tRNA^Glu^*, and *tRNA^Pro^*). The length range of the base of 13 protein-coding genes was 165 bp (ATP8) to 1824 bp (ND5). The majority of protein-coding genes (PCGs) initiated with ATG codon whereas *COI* gene initiated with GTG codon. The termination codons of PCGs were TAA (*ND1*, *COI*, *ATP6*, *COIII*, *ND4L*, *ND5*, and *ND6*), TAG (*ND2*, *ATP8*, and *ND3*) or an incomplete single T residue (*COII*, *ND4*, and *cyt b*). Twenty-two tRNA genes were interspersed among the rRNA and PCGs, with the size varying from 67 bp (*tRNA^Cys^*) to 76 bp (*tRNA^Leu^(UUR)*, *tRNA^Lys^*). There were two forms of *tRNA^Ser^* (UCN and AGY) and *tRNA^Leu^* (UUR and CUN). 12S rRNA (954 bp) and 16S rRNA (1678 bp) were located between *tRNA^Phe^* and *tRNA^Leu^* and separated by the *tRNA^Val^* gene.

There were two major noncoding regions. The origin of light-strand replication (OL), between the *tRNA^Asn^* and *tRNA^Cys^*, was 32 bp fragment and had the potential to fold into a stem-loop secondary structure. The displacement loop locus (D-loop; control region) was 923 bp sequence located between the *tRNA^Pro^* and *tRNA^Phe^* genes, with a high AT content of 66.74%. There were lesser AT repeats than *P. pingi* in D-Loop (9AT repeats cf. 13AT repeats; Li et al. 2013). There were 11 regions of gene overlaps totaling 29 bp (varying from 1 to 7 bp) and 11 intergenic spacer regions totaling 37 bp (varying from 1 to 13 bp).

To investigate the phylogenetic relationships between *P. retrodorsalis* and other cyprinid fishes, phylogenetic analyses were performed on the concatenated dataset of 28 PCGs (data were retrieved from GenBank, including outgroups *Silurus asotus*) with maximum likelihood method (Figure 1). The results showed that the fishes of genus *Percocypris* may have a close relationship with Schizothoracins (Schizothoracinae) compared to other fishes in the same family (Cyprinidae, fishes of subfamily Labeoninae, Barbinae, and Schizothoracinae), and it was consistent with that of previous phylogenetic study based on three mitochondrial gene sequences (*16S*, *COI*, and *cyt b*) and one nuclear marker (Rag2; Wang et al. 2013).

**Figure 1.**
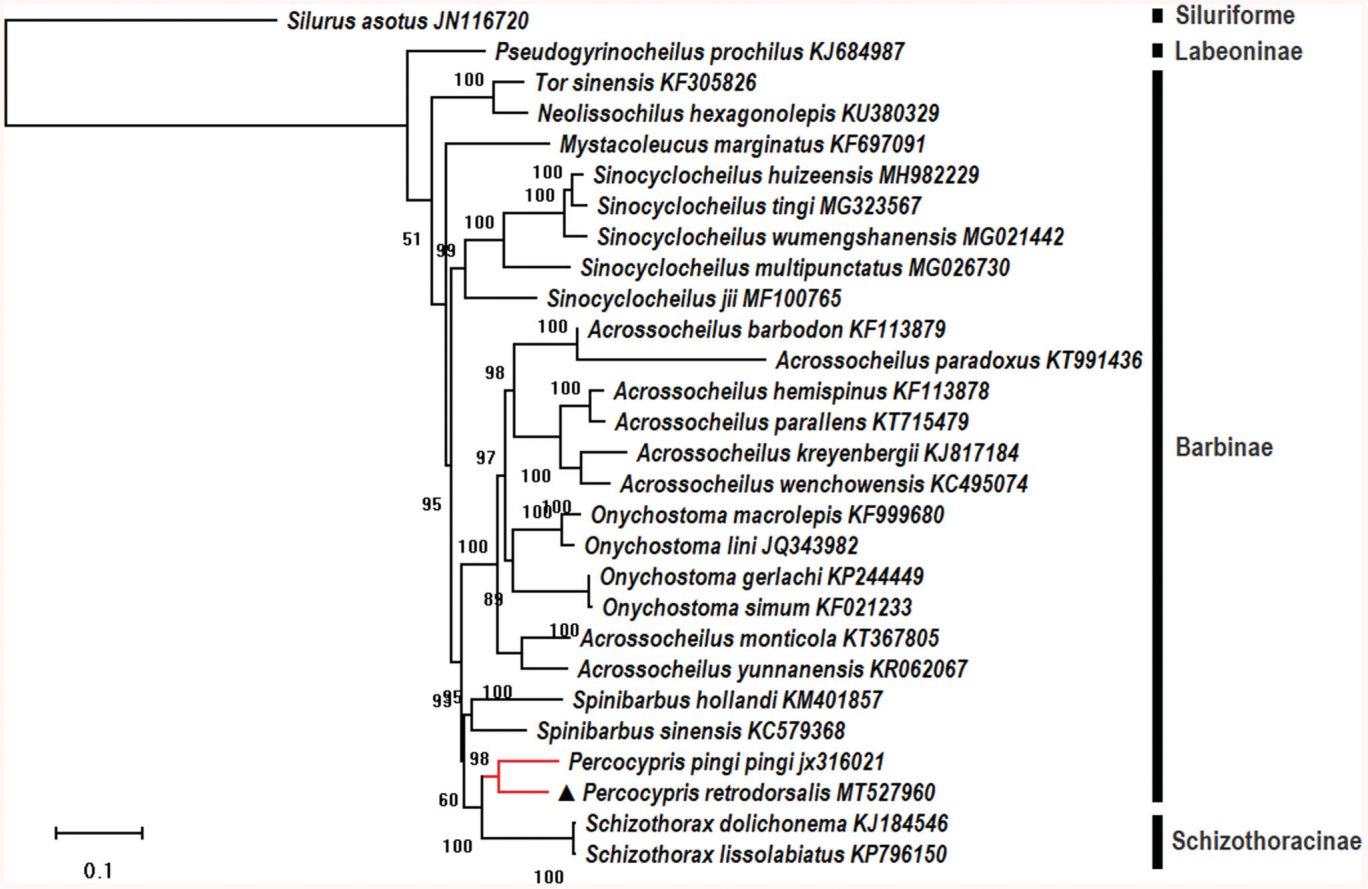
Molecular phylogenetic relationship among 28 fishes by maximum likelihood (ML) methods based on 13 protein-coding genes with 1000 bootstrap replications. This tree was drawn with setting of an outgroup (*Silurus asotus*).

## Data Availability

Data derived from public domain resources. The data that support the findings of this study are openly available in GenBank of NCBI at https://www.ncbi.nlm.nih.gov, reference number MT527960.
